# Sodium-Glucose Co-Transporter 2 Inhibitors: Mechanism of Action and Efficacy in Non-Diabetic Kidney Disease from Bench to Bed-Side

**DOI:** 10.3390/jcm13040956

**Published:** 2024-02-07

**Authors:** Aly M. Abdelrahman, Alaa S. Awad, Emaad M. Abdel-Rahman

**Affiliations:** 1 Department of Pharmacology & Clinical Pharmacy, College of Medicine and Health Sciences, Sultan Qaboos University, Alkhod 123, Oman; abdelrahman@squ.edu.om; 2 Division of Nephrology, University of Florida, Jacksonville, FL 32209, USA; alaa.awad@jax.ufl.edu; 3 Division of Nephrology, University of Virginia, Charlottesville, VA 22908, USA

**Keywords:** SGLT2i, non-diabetics, CKD, blood pressure, IgAN, FSGS, proteinuria, survival

## Abstract

Sodium-glucose cotransporter 2 inhibitors (SGLT2i) are currently available for the management of type 2 diabetes mellitus. SGLT2i acts by inhibiting renal SGLT2, thereby increasing glucosuria and lowering serum glucose. Recent trials are emerging supporting a role for SGLT2i irrespective of the diabetic status pointing towards that SGLT2i have other mechanisms of actions beyond blood sugar control. In this review, we will shed light on the role of this group of medications that act as SGLT2i in non-diabetics focusing on pre-clinical and clinical data highlighting the mechanism of renoprotection and effects of SGLT2i in the non-diabetic kidneys.

## 1. Introduction

Chronic kidney disease (CKD) is defined as persistently reduced estimated glomerular filtration rate (eGFR) <60 mL/min/1.73 m^2^, persistently elevated urine albumin excretion, or both for more than 3 months. As of 2022, CKD was shown to affect more than 800 million persons worldwide [1].

The prevalence of end-stage kidney disease (ESKD) as a consequence of progressive CKD and other kidney diseases has more than doubled in the USA from 389,592 to 807,920 between the years 2000 to 2020 [2] despite using current management. This continued increase in the prevalence of ESKD poses a huge burden on patients with kidney diseases and the health care system. Trials to add new medications to our armamentarium are much needed to improve the outcomes of patients with kidney diseases.

Recent trials evaluating a new group of medications that act on the sodium-glucose co-transporters (SGLTs) have shown promising results for kidney disease patients, especially in patients with diabetes mellitus (DM). Several trials confirmed the benefits of these medications in diabetic patients, yet little is known about their value in non-diabetic patients.

### 1.1. SGLT

SGLTs are transmembrane proteins that function to transport glucose and sodium across cell membranes in several tissues including the proximal renal tubules (PT). While SGLT1 contributes to the transport of glucose in the PT, it is SGLT2 that carries the burden of reabsorption of about 90% of filtered glucose in the PT. SGLT2 uses the electrochemical gradient of sodium ions to drive the transport of glucose against the concentration gradient [3].

In 2008, the US Food and Drug Administration mandated that SGLT2 inhibitors (SGLT2i) undergo outcome trials. These drugs were later approved to be among the newest classes of medications currently available for the management of type 2 DM [4,5]. SGLT2i act by inhibiting renal SGLT2, thereby increasing glucosuria and lowering serum glucose [6]. While their renoprotection in diabetics has been clearly documented [7], the potential for SGLT2i use and the mechanisms of nephroprotection [8] in non-diabetic chronic kidney disease is still not well defined. Yet, recent trials [9,10] weighed heavily in supporting the benefits of using SGLT2i irrespective of the diabetic status point towards SGLT2i having other mechanisms of action beyond blood sugar control.

In this review, we will shed light on the role of this group of medications that act as SGLTi in non-diabetic patients focusing on pre-clinical and clinical data highlighting the mechanism of renoprotection and effects of SGLT2i in the kidneys of patients without diabetes.

### 1.2. Mechanism of Renoprotection in Non-Diabetic Kidney beyond Glucose Control (Table 1)

The protective effects of SGLT2i can occur independently of blood sugar levels [11]. In addition to their antihyperglycemic effects, SGLT2i have potential actions on the cardiovascular and renal systems [12]. SGLT2i were shown to possess anti-inflammatory and antioxidant actions [13,14]. SGLT2i act directly on the endothelial system leading to decreased inflammatory cytokines and reactive oxygen species.

Changes noted in the tubule-glomerular (TG) feedback mechanism on using SGLT2i may contribute to the beneficial effects of these drugs on kidney function. SGLT2i cause natriuresis, promoting TG feedback and decreasing glomerular hyperfiltration [15].

Hawley et al. [16] showed that while canagliflozin significantly activates adenosine mono-phosphate-activated protein kinase (AMPK), dapagliflozin and empagliflozin effects on AMPK are significantly milder and require higher concentrations of these SGLT2i drugs. AMPK, when activated, regulates the energy status and energy hemostasis and stimulates autophagy, thus decreasing cellular stress and glomerular and tubular injury [17]. Furthermore, the increase in adenosine release by SGLT2i may lead to efferent arteriole vasodilatation [18]. These hemodynamic changes by SGLT2i may mediate an albuminuria-lowering action, further contributing to kidney protection [19]. Fioretto et al. [20] suggested that improvement of the lipid profile and body-weight reduction by SGLT2i may also help protect the kidneys.

Other effects of SGLT2i, not related to diabetes control, included the potential modest reduction in both systolic [21] and diastolic [22] blood pressure, mainly due to natriuresis and decreased circulating volume.

Furthermore, SGLT2i was shown to reduce serum uric acid. Zhao et al. [23] reviewed trials examining the effect of SGLT2i on serum uric acid in diabetic patients with at least 4 weeks of follow-up (*n* = 62 studies) and showed that SGLT2i significantly decreased serum uric acid compared with control, regardless of the SGLT2i drug used.

Yip et al. [24] showed similar results in reviewing 43 randomized controlled trials evaluating the effects of SGLT2i on serum uric acid in both patients with and without diabetes. They showed a reduction in serum uric acid by SGLT2i regardless of the diabetes status.

Rassaei et al. [25] studied the effect of SGLT2i in non-diabetic patients with CKD stages 3–5 (*n* = 78) and showed a reduction in serum uric acid from 6.4 to 5.6 mg/dl with an increase in fractional excretion of urate from 6.76% to 9.22% following the use of dapagliflozin.

**Table 1:** Renoprotective Mechanisms of Actions of SGLT2i.

-Antihyperglycemic -Anti-inflammatory → Decreasing inflammatory and reactive oxygen species. -Antioxidant -Promote tubule-glomerular feedback → Decrease glomerular hyperfiltration -Activate adenosine mono-phosphate-activated protein kinase → Decrease glomerular and tubular injury -Hemodynamic changes → Decrease albuminuria -Improve lipid profile -Reduce body weight -Natriuresis → Mild decrease in systolic and diastolic blood pressure -Attenuate renal ischemia-reperfusion injury -Decrease serum uric acid

## 2. SGLT2i and Non-Diabetic Kidney Dysfunction-Preclinical Experiments (Table 2)

### 2.1. Dapagliflozin

Dapagliflozin (10 mg/kg/day) for two days attenuated renal ischemia-reperfusion injury, improved renal function, reduced apoptotic cell death, and increased renal expression of hypoxia-induced factor 1 in C57BL/6 mice. The authors suggested that induction of hypoxia-induced factor 1 may play a role in renoprotection in this model [26]. It was also reported that dapagliflozin (1 and 10 mg/kg/day) for three weeks improved renal function and ameliorated renal fibrosis in a model of adenine-induced (0.2% diet) renal injury in C57BL/6J mice. This was associated with an increase in mitochondrial metabolism and fatty acid oxidation and a reduction of inflammation and oxidative stress. This study suggested that the renoprotective effect of dapagliflozin was related to mitochondrial protection due to inhibition of the transforming growth factor-β1/mitogen-activated protein kinase (TGF-β1/MAPK) pathway activation and a reduction of inflammation and oxidative stress [27]. Liu et al. [28] studied the effect of dapagliflozin on renal interstitial fibrosis induced by unilateral ureteral obstruction in C57BL/6J mice. They found that dapagliflozin (10 and 20 mg/kg/day) for two weeks improved renal function and renal fibrosis independent of direct blood glucose control. This was associated with reduced inflammation, apoptosis, oxidative stress, and mitochondrial injury in the kidneys. Deger et al. [29] showed that in Balb\c albino mice treated with cyclosporine A, dapagliflozin reduced oxidative stress, apoptosis, and histopathological damage in renal tissue caused by cyclosporin A. In C57BL/6N mice with protein-overload proteinuria induced by unilateral nephrectomy and injections of bovine serum albumin, dapagliflozin (1.5 mg/kg/day) treatment for twenty-three days reduced proteinuria and ameliorated podocyte dysfunction and loss and provided renal protection that was similar to lisinopril [30].

It was also shown that dapagliflozin has a renoprotective effect in a model of salt-induced hypertension and cardiorenal disease in Dahl salt-sensitive rats. In this study, dapagliflozin (0.1 mg/kg/day) for six weeks reduced albuminuria and attenuated renal inflammation and oxidative stress [31]. Firat et al. [6] demonstrated that dapagliflozin (0.1 mg/kg/day) for four weeks preserved the glomerular and mesangial structure and reduced renal oxidative stress in an iron-overload rat model. Chang et al. [32] showed that dapagliflozin (10 mg/kg/day) for six weeks attenuated doxorubicin-induced glomerular atrophy, renal fibrosis, and dysfunction and suppressed apoptosis and reactive oxygen species production in rats. In spontaneously hypertensive rats, dapagliflozin (10 mg/kg/day) for eight weeks reduced urinary albumin creatinine ratio but had no significant effect on serum creatinine levels or renal histological changes. However, transcriptome analysis showed that dapagliflozin therapy exerted protective effects on the renal tissues that were attributed to the alleviation of abnormal metabolism and inflammation [33]. Xuan et al. [7] showed that dapagliflozin (10 mg/kg/day) treatment for seven days reduced renal fibrosis in a unilateral ureteral obstruction rat model. On the contrary, in 5/6 (subtotally) nephrectomized rats, dapagliflozin (1 mg/kg/day) for twelve weeks did not attenuate heavy proteinuria, declining glomerular filtration rate, the extent of glomerulosclerosis, tubulointerstitial fibrosis or overexpression of the profibrotic cytokines, transforming growth factor-ß1 mRNA in the kidneys [34]. In subtotally nephrectomized rats, dapagliflozin (1 mg/kg/day) for eight weeks did not modify renal hemodynamic function nor did it attenuate proteinuria. In addition, dapagliflozin did not affect the increased glomerulosclerosis, increased glomerular collagen IV deposition, loss of glomerular capillaries, and increased infiltration of the tubule-interstitium by macrophages [35].

### 2.2. Canagliflozin

Canagliflozin (10 and 25 mg/kg/day) treatment for five weeks attenuated adenine-induced chronic kidney disease in rats. This action involved an anti-inflammatory effect as shown by reducing the inflammatory markers, tumor necrosis factor (TNF)-α, interleukin (IL)-6, and IL-1β as well as a reduction in oxidative stress [14]. In male C57BL/6 mice fed with 0.2% adenine, canagliflozin (10 mg/kg/day) treatment for two weeks, started after five weeks of an adenine diet, did not ameliorate renal damage; however, it reduced the accumulation of uremic toxins, including p-cresyl sulfate. The authors suggested that the lack of an effect on renal damage might be due to the administration period possibly being too short [36]. Moreover, in adenine-induced chronic kidney disease, treatment with canagliflozin (25 mg/kg/day) for four weeks failed to ameliorate the progressive loss of kidney function and there was no decreased interstitial area percentage, nor was there altered expression levels of genes related to fibrosis and inflammation. The authors concluded that canagliflozin dosage was not effective in a therapeutic setting of established non-diabetic CKD since canagliflozin treatment was started after four weeks of adenine treatment [37]. Canagliflozin (5 mg/kg/day) for one week was also shown to improve kidney function in isoprenaline-treated rats by stimulating antioxidant, anti-inflammatory, and anti-apoptotic signaling pathways [38]. In high-salt (8% sodium chloride) diet-induced Dahl salt-sensitive rats with hypertensive renal injury, canagliflozin (30 mg/kg/day) for twelve weeks attenuated the increase in blood pressure and ameliorated the associated hypertensive-induced renal injury. Canagliflozin increased sirtuin 3 expression, decreased epithelial-mesenchymal transition and oxidative stress, and inhibited renal fibrosis [39]. In addition, canagliflozin (10 and 30 mg/kg/day) for ten days reversed the biochemical and histopathological indices of cisplatin-induced nephrotoxicity in mice possibly through its anti-inflammatory and antioxidant effects [40]. Park et al. [41] also showed that in mice, canagliflozin (10 mg/kg/day) treatment for five days protected against cisplatin-induced acute kidney injury by activating adenosine monophosphate-activated protein kinase (AMPK) and autophagy in renal proximal tubular cells. Song et al. [42] also showed that in mice, canagliflozin (20 mg/kg/day) for eight days had a nephroprotective effect in cisplatin-induced nephrotoxicity. This effect mainly depends on Akt activation as well as reduced uptake of cisplatin in the kidneys. Moreover, canagliflozin (20 mg/kg/day) was effective in providing renal protection in unilateral ureteral occlusion and ischemia-reperfusion renal fibrosis mouse models [43]. In rats with membranous nephropathy, treatment with canagliflozin (10 mg/kg/day) for eight weeks decreased proteinuria and improved the hyperplasia of glomerular mesangial cells and stroma, and the thickening of the basement membrane and spiky structure. In addition, canagliflozin reduced renal immune complex deposition and improved podocyte injury. The authors concluded that canagliflozin exerts this renoprotective effect by reversing the imbalance in Helper T 1/Helper T 2 cells and restoring the autophagy of podocytes inhibited by the abnormal immunoglobulin G secretion from B-cells [44].

### 2.3. Empagliflozin

In a 5/6 nephrectomy rat model, a model of chronic kidney disease, empagliflozin (15 mg/kg/day) treatment for ninety-five days caused reduced proteinuria, improvement in creatinine clearance and renal interstitial fibrosis and glomerulosclerosis. The authors suggested two mechanisms of renoprotection: tubule-glomerular feedback-mediated effects and alterations of gene expression of key components of the complement system [8]. Lu et al. [45] also showed that empagliflozin (15 mg/kg/day) for six weeks had a beneficial effect on kidney function and morphology in the 5/6 nephrectomy rat model. They suggested that this beneficial effect might be due to an inhibition of CD206^+^CD68^+^ M2 macrophage polarization by targeting mammalian targets of rapamycin (mTOR) and mitophagy pathways and attenuating inflammatory signals from CD8^+^ effector T cells. Kim et al. [46] showed that empagliflozin (20 mg/kg/day) for three weeks in a rat model with salt-sensitive hypertension induced by uni-nephrectomy and 8% sodium chloride intake in Sprague Dawley rats decreased blood pressure and ameliorated renal inflammation. The beneficial renal protective effect of empagliflozin (10 mg/kg/day) for eight weeks was also shown in spontaneously hypertensive rats expressing human C-reactive protein and it was suggested that it was mediated by reduced renal lipid accumulation, inflammation, and oxidative stress [47]. In addition, empagliflozin (10 mg/kg/day) for four weeks reduced blood pressure and had a nephroprotective effect in cyclosporine A-induced nephropathy in rats [48]. In rats with angiotensin II-induced hypertension, empagliflozin (10 mg/kg/day) for two weeks prevented the development of renal fibrosis, an effect that was caused by a reduction in inflammatory infiltrates [49]. Reyes-Pardo et al. [50] showed also that empagliflozin (10 mg/kg/day) treatment for two weeks attenuated oxidative stress, proteinuria, and glomerular filtration rate reduction associated with angiotensin II infusion; thereby, reducing kidney damage development in a rat model of angiotensin II-dependent kidney damage. In hypertensive and proteinuric renin-transgenic (mRen2)27 rats with additional administration of N(ω)-nitro-L-arginine methyl ester (a nitric oxide synthase inhibitor), treatment with empagliflozin (3 and 10 mg/kg/day) dependently reduced proteinuria and induced protection for renal vasculopathy, glomerulopathy, and tubular degeneration [51]. The combination of the low dose (3 mg/kg/day) of empagliflozin and finerenone, a non-steroidal mineralocorticoid receptor antagonist, resulted in efficacious reduction in proteinuria, plasma creatinine and uric acid and renal lesions [51]. Empagliflozin (10 mg/kg/day), but not canagliflozin (30 mg/kg/day), for seven days was able to reduce renal tubular dilatation and necrosis in a renal ischemia-reperfusion injury model in rats. Empagliflozin did not have a significant effect on serum creatinine and blood urea nitrogen (BUN) levels while canagliflozin increased both parameters compared to the placebo-treated group. In addition, the increased renal kidney injury molecule (KIM)-1 expression and decreased urinary microRNA-26a excretion were alleviated by empagliflozin only [52]. Ala et al. [53] also showed that empagliflozin (10 mg/kg/day) for two days attenuated renal ischemia-reperfusion injury in rats that was associated with reduced oxidative stress, inflammation, and apoptosis. In C57/BL6 mice subjected to renal ischemia-reperfusion injury, empagliflozin (1 mg/kg) given twice protected against renal injury as shown by decreased serum levels of creatinine, attenuated tubular damage, reduced inflammatory markers, and inhibited apoptosis [54]. Ge et al. [55] showed that empagliflozin (70 mg/kg/day in the chow) for four weeks reduced podocyte lipotoxicity, prevented renal lipid accumulation, and improved renal function in a mouse model of Alport syndrome [55]. In a mouse model with vascular calcification induced by an oral high-phosphorus diet following a 5/6 nephrectomy in Apo E−/− mice, empagliflozin (10 mg/kg/day) treatment for eight weeks improved renal function [56]. Empagliflozin (10 mg/kg/day) treatment for fourteen days did not affect chronic kidney disease progression in oxalate-related nephrocalcinosis in C57BL/6N mice. This was demonstrated as empagliflozin had no effect on reduced glomerular filtration rate, crystal deposition, tubular injury, and markers of interstitial fibrosis [57]. In three models of chronic kidney disease, fawn-hooded hypertensive rats, uni-nephrectomized salt-loaded rats, and rats with Goldblatt hypertension (two-kidney, one-clip 2K1C), empagliflozin (10 mg/kg/day) for eight weeks did not provide renoprotection because it did not ameliorate proteinuria, elevated plasma urea and creatinine, oxidative stress, or inflammation [58].

### 2.4. Ipragliflozin

Ipragliflozin (0.3 and 0.1 mg/kg/day) treatment for four weeks was shown to have a renoprotective effect that was independent of plasma glucose levels and urinary glucose excretion in adenine-induced (0.25% *w*/*w* in the diet) chronic kidney disease in C57BL/6JJcl mice. The anti-inflammatory and antioxidant effect of ipragliflozin might have contributed to the protective effect [59]. In addition, ipragliflozin (1 mg/kg/day) for twelve weeks improved the pathogenesis of chronic kidney disease by reducing ectopic lipid deposition in renal tubules, endoplasmic reticulum stress in a mouse (FLS-*ob*/*ob)* model of non-alcoholic steatohepatitis [60]. Ipragliflozin (0.04%) monotherapy during eight weeks of a high-salt diet did not improve renal glomerulosclerosis or creatinine clearance in Dahl salt-sensitive rats. However, the combination therapy of ipragliflozin and losartan significantly ameliorated glomerulosclerosis compared with ipragliflozin or losartan monotherapy [61].

In conclusion, preclinical trials showed that dapagliflozin, canagliflozin, empagliflozin, or ipragliflozin had renoprotective effects in various animal models of acute kidney injury and chronic kidney diseases.

**Table 2:** Effects of SGLT2i on Experimental Animals.

Drug	Animal	Effects of SGLT2i	Ref.
**Dapagliflozin**	-C57BL/6 mice with renal-reperfusion injury	-Attenuated renal ischemia-reperfusion injury, improved renal function, reduced apoptotic cell death and increased renal expression of hypoxia induced factor 1	[26]
	-C57BL/6J mice with adenine (0.2% diet) -induced renal injury	-Improved renal function and ameliorated renal fibrosis, increased mitochondrial metabolism and fatty acid oxidation, reduction of inflammation and oxidative stress.	[27]
	-Unilateral ureteral obstruction in C57BL/6J mice	-Improved renal function and renal fibrosis independent of direct blood glucose control	[28]
	-Balb\c albino mice treated with cyclosporine A	-Reduced oxidative stress, apoptosis, and histopathological damage in renal tissue	[29]
	-C57BL/6N mice with protein-overload proteinuria induced by unilateral nephrectomy and injections of bovine serum albumin	-Reduced proteinuria and ameliorated podocyte dysfunction and loss and provided renal protection	[30]
	- Dahl salt sensitive rats with salt-induced hypertension and cardiorenal disease	-Reduced albuminuria and attenuated renal inflammation and oxidative stress	[31]
	-Iron-overload rat model	- Preserved the glomerular and mesangial structure and reduced renal oxidative stress	[5]
	-Rats with doxorubicin induced glomerular atrophy	-Attenuated glomerular atrophy, renal fibrosis, and dysfunction	[32]
	-Spontaneously hypertensive rats	-Reduced urinary albumin creatinine ratio but had no significant effect on serum creatinine levels or renal histological changes	[33]
	-Rats with unilateral ureteral obstruction	-Reduced renal fibrosis	[7]
	-Rats with 5/6 (subtotally) nephrectomized	-**Did not** attenuate heavy proteinuria, declining glomerular filtration rate, the extent of glomerulosclerosis or tubulointerstitial fibrosis	[34]
	-Rats with subtotally nephrectomized	-**Did not** modify renal hemodynamic function nor attenuated proteinuria	[35]
**Canagliflozin**	-Rats with adenine-induced chronic kidney disease	-Attenuated adenine induced chronic kidney disease, anti-inflammatory effect as well as reduction in oxidative stress	[13]
	-Male C57BL/6 mice fed with 0.2% adenine	-**Did not** ameliorate renal damage, however it reduced the accumulation of uremic toxins including p-cresyl sulfate	[36]
	-Rats with adenine-induced chronic kidney disease	-**Failed** to ameliorate the progressive loss of kidney function and there was no decreased interstitial area percentage, nor was there altered expression levels of genes related to fibrosis and inflammation	[37]
	-Rats, isoprenaline-treated	-Improve kidney function by stimulating antioxidant, anti-inflammatory and anti-apoptotic signaling pathways	[38]
	-Dahl salt-sensitive rats with high-salt diet-inducing hypertensive renal injury.	-Attenuated the increase in blood pressure and ameliorated the associated hypertensive-induced renal injury, decreased epithelial-mesenchymal transition and oxidative stress and inhibited renal fibrosis	[39]
	-Mice with cisplatin-induced nephrotoxicity.	-Reversed the biochemical and histopathological indices of possibly through its anti-inflammatory and antioxidant effects	[40]
	-Mice with cisplatin-induced nephrotoxicity.	-Protected against cisplatin-induced acute kidney injury by activating adenosine monophosphate-activated protein kinase and autophagy in renal proximal tubular cells	[41]
	-Mice with cisplatin induced nephrotoxicity.	-Nephroprotective effect by Akt activation, reduced uptake of cisplatin in the kidneys.	[42]
	-Mice with unilateral ureteral occlusion and ischemia-reperfusion renal fibrosis	-Renal protection	[43]
	-Rats with membranous nephropathy	-Decreased proteinuria and improved the hyperplasia of glomerular mesangial cells and stroma, the thickening of basement membrane and spiky structure.	[44]
**Empagliflozin**	-Rats with 5/6 nephrectomy	-Reduced proteinuria, improved in creatinine clearance and renal interstitial fibrosis and glomerulosclerosis.	[7]
	-Rats with 5/6 nephrectomy	-beneficial effect on kidney function and morphology due to an inhibition of CD206+CD68+ M2 macrophage polarization by targeting mammalian target of rapamycin (mTOR) and mitophagy pathways and attenuating inflammatory signals from CD8+ effector T cells.	[45]
	-Sprague Dawley rats with uni-nephrectomy and salt sensitive hypertension.	-Decreased blood pressure and ameliorated renal inflammation.	[46]
	-Spontaneously hypertensive rats	-Beneficial renal protection by reducing renal lipid accumulation, inflammation and oxidative stress	[47]
	-Rats with cyclosporine A induced nephropathy	-Reduced blood pressure	[48]
	-Rats with angiotensin II induced hypertension	-Prevented the development of renal fibrosis by reducing inflammatory infiltrates	[49]
	-Rats with angiotensin II dependent kidney damage	-Reduced kidney damage by attenuating oxidative stress, proteinuria and glomerular filtration rate reduction associated with angiotensin II infusion	[50]
	-Hypertensive and proteinuric renin-transgenic (mRen2)27 rats with additional administration of nitric oxide synthase inhibitor	-Reduced proteinuria and induced protection for renal vasculopathy, glomerulopathy, and tubular degeneration	[51]
	-Rats with renal ischemia-reperfusion injury	-Reduced renal tubular dilatation and necrosis	[52]
	-Rats with renal ischemia-reperfusion injury	-Attenuated renal injury with reduced oxidative stress, inflammation an apoptosis	[53]
	-C57/BL6 mice subjected to renal ischemia-reperfusion injury	-Protected against renal injury, attenuated tubular damage, reduced inflammatory markers and inhibited apoptosis	[54]
	-Mouse model of Alport syndrome	-Reduced podocyte lipotoxicity prevented renal lipid accumulation and improved renal function	[55]
	-Apo E−/− mice with vascular calcification and 5/6 nephrectomy	-Improved renal function	[56]
	-C57BL/6N mice with oxalate-related nephrocalcinosis	-**Did not** affect chronic kidney disease progression in oxalate-related nephrocalcinosis	[57]
	-Fawn-hooded hypertensive rats-Uni-nephrectomized salt-loaded rats-Rats with Goldblatt hypertension	-**Did not** provide renoprotection because it did not ameliorate proteinuria, elevated plasma urea and creatinine, oxidative stress or inflammation	[58]
**Ipragliflozin**	-C57BL/6JJcl mice with adenine induced chronic kidney disease	-Renoprotective effect that was independent from plasma glucose levels and urinary glucose excretion	[59]
	-Mouse (FLS-*ob*/*ob*) model of non-alcoholic steatohepatitis	-Improved the pathogenesis of chronic kidney disease by reducing ectopic lipid deposition in renal tubules, endoplasmic reticulum stress	[60]
	-Dahl Salt sensitive rats	-**Did not** improve renal glomerulosclerosis or creatinine clearance	[61]

## 3. SGLT2 and Non-Diabetic Kidney Dysfunction-Clinical Trials (Table 3)

### 3.1. Effects of SGLT2i on CKD

Several original trials and meta-analysis studies were conducted to elicit the effects of SGLT2i on patients with CKD. These studies included both patients with and without DM.

**Table 3:** Renal Beneficial Effects of SGLT2i in Non-Diabetic Patients.

- Delay progression of CKD - Role in management of IgA nephropathy - Potential role in the management of focal segmental glomerulosclerosis - Decrease blood pressure - Decrease proteinuria - Improve renal-related survival

**Figure 1:**
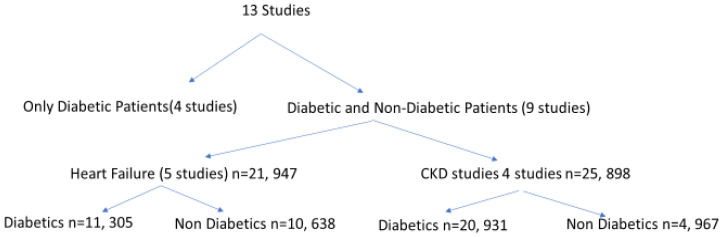
Diabetics versus non-diabetics in metanalysis.

We looked to tease the effect of SGLT2i on non-diabetics in these metanalyses. A recent study [62] underwent a systemic review and metanalysis of large placebo-controlled trials using SGLT2i and identified 13 studies with 90,413 participants (Figure 1). Of those, the number of non-diabetic patients was 15,605/47,845 participants (32.6%).

Of the four CKD studies, two studies involved most of the non-diabetic patients. The study by Heerspink et al. [63] (DAPA-CKD) included 1398/4304 (32.5%) non-diabetic patients while the EMPA-CKD trial [10] had 3569/6609 (54.0%) with no DM. Their mean baseline eGFR ranged from 37–56 mL/min/1.73 m^2^ with a follow-up of 1.3–2.6 years.

The study by Heerspink et al. [63] was prematurely stopped as dapagliflozin proved its efficacy versus placebo over a median of 2.4 years. They showed a reduced risk of a composite of a sustained decline in the estimated GFR of at least 50%, ESKD, or death from renal or cardiovascular causes with dapagliflozin than with placebo. Similarly, empagliflozin showed a similar superiority over placebo in decreasing the composite progression of kidney disease [10]. In both studies, the diabetic status, the baseline eGFR, and the primary cause of CKD had no impact on the renal outcomes.

Four out of the 5 heart failure studies included non-diabetic patients [64,65,66,67]. Their mean eGFR ranged from 62 to 68 mL/min/1.73 m^2^, with a follow-up ranging between 0.8–and 2.2 years. Analyzing these studies showed that SGLT2i decreased the risk of CKD progression regardless of the presence or absence of DM, baseline eGFR, or the primary etiology of CKD.

A year later, Rassaei et al. [25] added five more studies to the prior metanalysis and studied the impact of SGLT2i on renal parameters focusing on non-diabetic patients. Out of 46 full texts, they reviewed 7 studies in depth [9,36,64,68,69,70,71]. Studies reviewed had a follow-up period ranging from 6 weeks to 2.4 years; 6 trials used dapagliflozin [9,35,64,68,69,70] while one trial used empagliflozin [71]. Their review demonstrated that SGL2i had a reno-protective effect as demonstrated by delaying the reduction of eGFR and decreasing urine albumin/creatinine ratio (UACR). Vart et al. [72] further suggested that adding RAAS blockers to SGLT2i in non-diabetic albuminuric patients may increase kidney failure-free survival. While in one study [59], dapagliflozin was shown to cause a reversible decrease in eGFR in six weeks after the washout period following stopping the drug, another study [35] failed to show that dapagliflozin benefits patients who had preserved kidney function.

### 3.2. Effects of SGLT2i on Glomerular Disorders

#### 3.2.1. IgA Nephropathy (IgAN)

Wheeler et al. [69] analyzed the DAPA-CKD study based [9] on the primary etiology of CKD. The studied outcome was the effects of dapagliflozin on sustained decline in eGFR of 50% or more, ESKD, or death from a kidney disease-related or cardiovascular cause. The study included 270 participants with investigator-reported IgAN (confirmed by renal biopsy in 94% of the participants). Only 14.1% of the cohort had DM. Participants receiving dapagliflozin (*n* = 137) and those receiving placebo (*n* = 133) were followed for a median of 2.1 years (0.025–3.2 years). Mean eGFR and median UACR were 44.3 (12.4) mL/min/1.73 m^2^ and 889.5 (557.5–1472.0) mg/g and 43.2 (12.0) mL/min/1.73 m^2^ and 902.5 (500.5–1633.0) mg/g in the dapagliflozin and placebo, respectively. The study showed the superiority of dapagliflozin over placebo on primary composite (*p* = 0.005) and secondary kidney-specific outcomes (*p* = 0.002). There was a 76% reduction in ESKD and a >50% reduction in eGFR decline and renal death. These effects did not differ based on eGFR or UACR with an annual decline in eGFR less in the dapagliflozin group versus placebo (−2.2 +/− 0.5 vs. −4.6 +/− 0.47 mL/min/1.73 m^2^).

Similar results were found after analyzing the EMPA-KIDNEY trial [10] with 817 patients in the study having IgAN. Empagliflozin reduced the kidney endpoint of IgAN patients (progression of CKD disease) by about 30% vs. placebo. Combining results of these 2 studies showed a 51% decrease in CKD progression in patients with IgAN [73].

Dong et al. [74] followed a cohort of Chinese patients with biopsy-proven IgAN for three and six months after being treated with SGLT2i. Most of the IgAN cohort were non-diabetic (81.7%). The authors observed a significant reduction in proteinuria in their patient cohort independent of immunosuppressive agents and baseline eGFR and proteinuria levels, again confirming the role of SGLT2i in the management of IgAN.

#### 3.2.2. Focal Segmental Glomerulosclerosis (FSGS)

Rajasekeran et al. studied [35] the effect of eight weeks of dapagliflozin on GFR in humans (*n* = 10) and in experimental FSGS. The secondary endpoints were related to changes in renal hemodynamic function, proteinuria, and blood pressure. They noted no statistical difference in PAH-based GFR. When stratified into two subgroups based on GFR, the researchers noted a significant decrease in GFR in the group of patients with GFR > 90 m/min/1.73 m^2^. They further showed that dapagliflozin failed to modify kidney hemodynamics or attenuate proteinuria.

Two years after the prior study, Cherney et al. [68] performed a short-term crossover, randomized controlled trial (DIAMOND) at six hospitals in three countries. Their goal was to investigate the efficacy of SGLT2i in proteinuric kidney disease without diabetes. Eleven patients with FSGS were randomly assigned (1:1) to receive a placebo followed by dapagliflozin 10 mg per day or vice versa. Each treatment period lasted six weeks with a six-week washout period in between. The primary outcome was the effect of dapagliflozin on proteinuria and other hemodynamic parameters. While the researchers demonstrated a nonsignificant reduction in proteinuria compared with placebo, GFR was temporarily decreased during the six weeks of dapagliflozin.

A larger study analyzing patients with biopsy-proven FSGS (*n* = 104) in the DAPA-CKD trial [63] showed a reduced rate of eGFR decline versus placebo but failed to show any statistically significant difference between the drug and the placebo group [75]. It is postulated that this negative result could be attributed to the small number of events (4 primary outcomes in dapagliflozin versus 7 in the placebo group).

Thus, data remains non-conclusive as to the role of SGLT2i in managing patients with FSGS. The lack of long-term data and a larger study population to address the potential efficacy of SGLT2i in FSGS leave a knowledge gap that begs for further studies.

### 3.3. Effects of SGLT2i on Blood Pressure

Miyata et al. [11] reviewed the mechanisms of decreasing blood pressure upon using SGLT2i. They suggested that the decrease in plasma volume as a result of their diuretic and natriuretic effects, weight loss, and the anti-inflammatory action of these drugs may contribute to the anti-hypertensive effects. Other mechanisms identified that may contribute to the SGLT2i effects on blood pressure were the noted improvement in arterial stiffness and endothelial function [21,76].

While the effect of SGLT2i in lowering blood pressure in diabetics is well established [77], studies of the role of SGLT2i in lowering blood pressure in non-diabetics did not show the same effect [36,68]. Cherney et al. [68] studied non-diabetic patients (*n* = 53, GFR > 25 mL/min, proteinuria 500–3500 mg/g) comparing dapagliflozin effects on blood pressure versus placebo over six weeks. Patients who started on dapagliflozin (*n* = 27) were switched to a placebo, and patients on a placebo (*n* = 26) were switched to receive dapagliflozin. The researchers showed no change in systolic (SBP) and diastolic (DBP) blood pressure with no hypoglycemic episodes. The decrease in body weight with dapagliflozin was 1.5 kg (*p* = 0.046).

Zanchi et al. [78] performed a randomized control study on non-diabetic normotensive patients (*n* = 39) comparing the effect of empagliflozin (*n*-26) versus placebo (*n* = 13) on blood pressure. Their results were different than the prior studies where empagliflozin was shown to decrease SBP/DBP (−5 ± 7 and −2 ± 6, respectively) over a one-month study period. Similarly, Bays et al. [79] underwent a randomized, double-blinded study comparing the canagliflozin effect in three different dosages on blood pressure in non-diabetics versus placebo (*n* = 376). At the end of the study (12 weeks), the researchers demonstrated a decrease in blood pressure in both canagliflozin and placebo groups. While the reduction in SBP was more in the canagliflozin groups (−2.0 to −3.3 vs. −1.4 mmHg), the reduction in DBP was more in the placebo group (−1.8 vs. −0.7 to −1.4 mmHg). Diaz-Cruz et al. [80] were also able to show a decrease in both SBP and DBP on using dapagliflozin in patients who were pre-diabetics.

### 3.4. Effects of SGLT2i on Nephrotic Proteinuria

Kalay et al. [81] reviewed studies examining the effects of SGLT2i on patients with nephrotic range proteinuria and identified nine studies. While most patients in these studies were diabetics, two studies included non-diabetic patients with FSGS [82,83].

Boeckhaus et al. [82] reported 2 cases with FSGS where SGLT2i reduced the UACR ratio by 84% at 11 months in one patient and by 18% at 3 months in another. The same data was reported in another study [83] on a patient with FSGS where UACR was decreased by 61% after one month and by 37% after 9 months of using SGLT2i.

## 4. Effects of SGLT2i on Survival

Awad et al. [84] reviewed randomized clinical trials (12 studies) focusing on the effect of SGLT2i on mortality in both diabetic and non-diabetic patients. While mortality risks were decreased in both groups, it was not statistically significant in the non-diabetic patients (RR = 0.93, 95% CI 0.70–1.23).

Heerspink et al. [9] showed that dapagliflozin decreased the risk of both renal and cardiovascular mortality, and when RAAS blockers were added to dapagliflozin, patients’ survival was further increased. More recently, McEwan et al. [85] used a model analysis on the DAPA-CKD trial and showed that dapagliflozin may reduce all-cause mortality.

## 5. SGLT2i Side Effects

While using SGLT2i was not associated with hypoglycemia in non-diabetic patients, several significant side effects were noted when using these drugs.

Hypotension secondary to diuresis [9], natriuresis, and volume depletion has been reported. Furthermore, excessive diuresis may impact poorly on patients’ quality of life. Urinary tract infections (UTIs) mainly secondary to yeast infections have been associated with the use of SGLT2i [86]. UTIs can range from mild to severe infections. A more severe form of infection, genital mycotic infection, has been described in diabetics, but not in non-diabetics [87].

Other side effects were suggested to be associated with SGLT2i as bone fractures, amputations, and malignancies, but results are controversial [88].

## 6. Future Directives

The addition of newer agents that showed benefits in managing patients with kidney diseases to SGLT2i may be beneficial. Zhang et al. [89] performed a systemic literature search of randomized control trials comparing mineralocorticoid receptor antagonists (finerenone), SGLT-2i, and glucagon-like peptide-1 receptor agonists (GLP-1 RA) in diabetics with CKD. They identified 18 studies (*n* = 51,496) that demonstrated that while SGLT2i significantly decreased the risk of renal events compared with finerenone and GLP-1 RA, their cardiac outcome effects were comparable.

Trials comparing additive effects of SGLT2i with GLP-1 RA, mineralocorticoid, or endothelin receptor antagonist to SGLT2i alone in both diabetics and non-diabetics may prove to be very helpful in improving renal outcomes.

## 7. Conclusions

SGLT2i play a major role in managing non-diabetic patients with kidney diseases. This group of drugs can achieve their role through different mechanisms unrelated to glycemic control. More studies looking at the effects of SGLT2i on different renal pathologies causing CKD, examining the effects of SGLT2i alone or in combination with other therapeutic groups, their possible side effects, and their mechanism of action are very much needed.

It is important to note that many studies reviewed were small studies, open labeled with little reports on control variables that may affect the outcomes of the study. It is crucial to have more well-designed randomized placebo-controlled studies.
